# Skin hydration dynamics investigated by electrical impedance techniques in vivo and in vitro

**DOI:** 10.1038/s41598-020-73684-y

**Published:** 2020-10-14

**Authors:** Maxim Morin, Tautgirdas Ruzgas, Per Svedenhag, Christopher D. Anderson, Stig Ollmar, Johan Engblom, Sebastian Björklund

**Affiliations:** 1 Biofilms – Research Center for Biointerfaces, Malmö University, Malmö, Sweden; 2 Department of Biomedical Science, Faculty of Health and Society, Malmö University, Malmö, Sweden; 3 SciBase AB, Sundbyberg, Sweden; 4 Department of Biomedical and Clinical Sciences, Linköping University, Linköping, Sweden; 5 Department of Clinical Science, Intervention and Technology, Karolinska Institutet, Stockholm, Sweden

**Keywords:** Biophysics, Medical research, Electrochemistry, Electrical and electronic engineering, Biological physics

## Abstract

Skin is easily accessible for transdermal drug delivery and also attractive for biomarker sampling. These applications are strongly influenced by hydration where elevated hydration generally leads to increased skin permeability. Thus, favorable transdermal delivery and extraction conditions can be easily obtained by exploiting elevated skin hydration. Here, we provide a detailed in vivo and in vitro investigation of the skin hydration dynamics using three techniques based on electrical impedance spectroscopy. Good correlation between in vivo and in vitro results is demonstrated, which implies that simple but realistic in vitro models can be used for further studies related to skin hydration (e.g., cosmetic testing). Importantly, the results show that hydration proceeds in two stages. Firstly, hydration between 5 and 10 min results in a drastic skin impedance change, which is interpreted as filling of superficial voids in skin with conducting electrolyte solution. Secondly, a subtle impedance change is observed over time, which is interpreted as leveling of the water gradient across skin leading to structural relaxation/changes of the macromolecular skin barrier components. With respect to transdermal drug delivery and extraction of biomarkers; 1 h of hydration is suggested to result in beneficial and stable conditions in terms of high skin permeability and extraction efficiency.

## Introduction

The skin provides an attractive route for drug delivery due to avoidance of first pass metabolism^1^. Similarly, considering that the skin surface is easily accessible, it also serves as an attractive sampling site for non-invasive extraction of low molecular weight (LMW) biomarkers (< 500 Da), such as biomarkers related to inflammation and skin cancer^2^. However, for transdermal drug delivery and non-invasive sampling to be successful, the drug or the biomarker has to pass the outermost layer of the epidermis, the stratum corneum (SC), which forms the main barrier of the skin^3^. Due to its important role as a barrier, the SC layer has been extensively studied over the years and different strategies aimed at increasing its permeability have been investigated^1^. Some common strategies include, for example, chemical penetration enhancers^4^ and physical techniques such as microneedles^5^, electroporation^6^, iontophoresis^7^, and more^1^. Further, numerous studies, performed both in vivo and in vitro, have shown that the degree of SC hydration has a strong impact on its barrier properties^8–12^. Strikingly, close to full hydration of the SC (e.g. by skin occlusion) leads to enhanced permeability of both hydrophilic and hydrophobic molecules across the skin barrier, which implies that the skin permeability can be tuned by regulating the SC hydration^10,13^. Importantly, at fully hydrated conditions, the diffusion characteristics within the SC barrier are relatively stable and favorable for enhanced transdermal drug delivery or extraction of molecules originating from the skin^10,13^. Thus, characterization of the degree of skin hydration is of great interest for development of successful transdermal delivery applications and non-invasive monitoring of LMW biomarkers. Moreover, hydration of the SC is important for its flexibility, softness, and pliability, which are important parameters to optimize so that the SC can tolerate external physical stress^14,15^. Similarly, a few studies have emphasized the importance of sufficient amount of water for biochemical and enzymatic reactions to take place in the skin barrier, which is most likely crucial for maintaining a healthy status of the skin^16–18^. The degree of skin hydration can be regulated by application of a topical formulation where the skin becomes hydrated either by water supplied as a formulation ingredient or as an endogenous by-product from transepidermal water loss (TEWL) when the formulation occludes the skin surface^19^.

Taken together, in order to optimize the permeation of drugs and ensure that extraction of biomarkers occurs in an accurate, controlled, and reproducible manner, it is important to consider the hydration of the SC after application of a formulation, dressing, or sampling patch. Therefore, increased understanding of the hydration process, as well as determination of the time required to reach full skin hydration, is highly important. Ever since the pioneering work by Blank in the early 1950s, highlighting factors that influence the water content of the skin^14,15^, the effect of hydration on the skin barrier has been relentlessly investigated by various methods with continuously updated technical features^9,11,12,20–29^. These studies have provided both molecular and macroscopic information on the various effects of hydration on the SC properties and function.

For example, Bouwstra et al. explored the structural properties of the lipid lamellar organization of excised SC sheets with SWAXS (small- and wide-angle X-ray scattering) and concluded that neither the lateral crystal structure nor the transverse lamellar repeat distance were affected by hydration^21,22^. Similarly, Mak et al. performed FTIR (Fourier transform infrared spectroscopy) experiments to probe the C–H asymmetric stretching frequency (2920 cm^−1^) and concluded that SC hydration does not lead to an overall increase of the SC lipid disorder^12^. In contrast, Alonso et al. utilized an ESP (electron spin resonance) method with added stearic acid (C18) spin labels and probed the correlation time of the C5, C12, and C16 positions of the alkyl chain and concluded that hydration leads to increased lipid fluidity, in particular for the positions close to the lipid headgroups^11^. In support for this, Silva et al. employed relaxation and wide line ^1^H NMR (nuclear magnetic resonance) methods and concluded that the fraction of fluid lipids increased when SC was hydrated from a dry state to a moderately hydrated state, while no further effect was observed at higher water contents^26^. Following this, Björklund et al. employed solid-state NMR with an advanced combination of polarization transfer schemes, referred to as PT ssNMR (polarization transfer solid-state NMR)^30^, providing detailed resolution of the SC molecular segments, and concluded that the ratio between fluid and solid lipid segments (i.e., CH_2_ and CH_3_ lipid carbons) increases with hydration^20^. Further, a drastic increase of the dynamics of molecular segments found in the keratin filaments was observed between 80 and 85% relative humidity; from a completely rigid keratin structure to a structure with both solid and mobile parts of the keratin filaments^20,31^. In line with this finding, that hydration leads to increased keratin mobility, Norlén et al. concluded that the dimensions of SC pieces swell about 26% in thickness and 4% in width when comparing the dry state with a hydrated state (i.e., after 90 min soaking in pure water) by employing a CLSM (confocal laser scanning microscopy) method^27^. Strikingly, this distinct swelling in the transverse (thickness) direction of the corneocytes leads to a drastic unfolding of the extracellular lipid domains, which has been clearly illustrated by CEM (cryo-electron microscopy) images on dehydrated, normal hydrated, and fully hydrated SC pieces^23,28^. However, even at full hydration, the corneocytes in the deepest regions of the SC do not seem to swell, but rather remain in a similar morphology as the corneocytes of dry SC in the corresponding cell layer^23,28^. Another interesting characteristic of fully hydrated skin is the presence of intercellular aqueous inclusions (i.e., aqueous pools of varying sizes) in the SC, which have been observed in several studies utilizing different microscopy techniques^23–25,29^.

Taken together, the mentioned studies comprehensively describe the effect of hydration on the SC molecular and macroscopic properties at static hydration levels when the SC tissue is in equilibrium, either with a specific relative humidity (RH) or prepared with a defined water content. However, the dynamics of the hydration process remain less studied; in particular under in vivo conditions. A better understanding of the hydration dynamics of the skin barrier is of high importance for several reasons, such as better understanding of how transdermal drug delivery is affected by different formulations and dressings, how to optimize non-invasive extraction of low molecular weight biomarkers from skin, or how to support claims related to improved skin hydration for the cosmetic industry^32^. In this work, the dynamics of skin hydration is assessed with electrical impedance spectroscopy (EIS) techniques. In general, changes of the electrical properties of SC are strongly dependent on the hydration state, which implies that EIS is a very suitable methodology to study the hydration process^9,32,33^. Based on both in vivo and in vitro studies, it is established that the electrical impedance of skin resides foremost within the SC, while the impedance of the underlying viable layers is orders of magnitude lower^34^. Notably, changes of the degree of SC hydration can have a dramatic influence on its conductive and capacitive properties^9^. Moreover, the resistance of skin is directly correlated to its barrier capacity, both of which depend on the hydration degree of SC^9,10^. This further implies that changes in the skin barrier function caused by hydration can be studied by means of EIS due to its high sensitivity to the water content.

**Figure 1:**
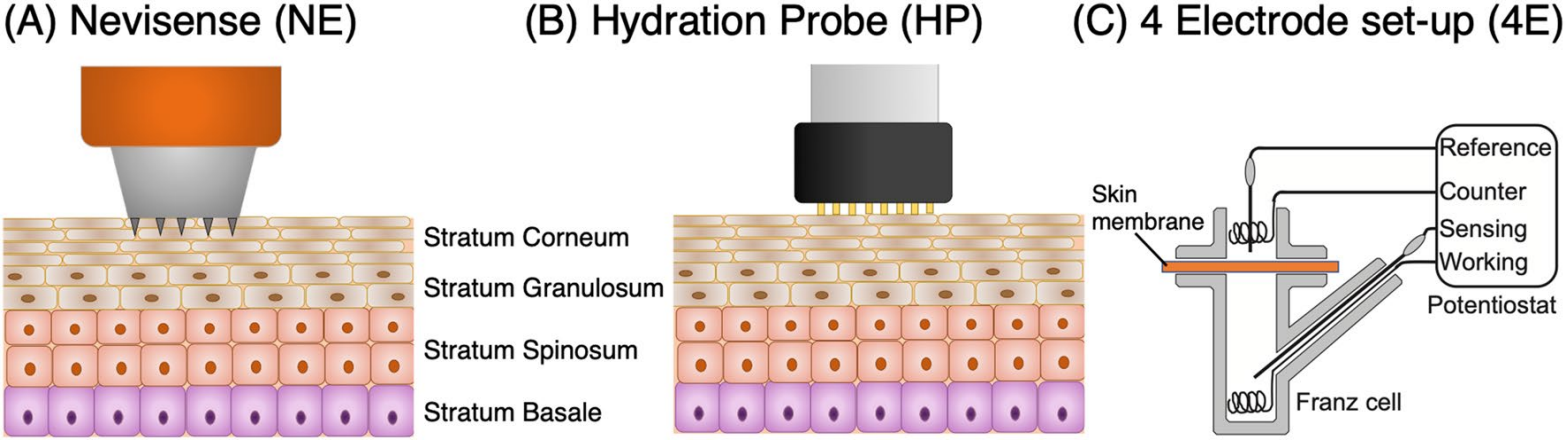
Electrical impedance spectroscopy (EIS) techniques used in this work to study the effect of hydration on the stratum corneum (SC) electrical impedance properties.

The aim of this work was to gain a better understanding of the dynamics of skin hydration. In particular, our ambition was to determine the hydration rate and the time required to reach a stable hydration level following a specific hydration treatment. The hydration process was investigated with EIS both in vivo and in vitro, using alternative methods, i.e., Nevisense (NE, SciBase AB, Sweden), DermaLab Hydration Probe (HP, Cortex Technology, Denmark) and a four-electrode set-up mounted in a conventional Franz cell (4E), see Fig. 1. The study was designed to allow for correlation analysis between the different methods and also between in vitro and in vivo results. Finally, the hydration process was investigated in vitro at different temperatures to elucidate the interplay between thermal and hydration effects on the electrical properties of the SC barrier.

## Materials and methods

### Materials

#### Chemicals

Sodium chloride (NaCl), sodium phosphate dibasic dihydrate (Na_2_HPO_4_·2H_2_O) and monobasic potassium phosphate (KH_2_PO_4_) were purchased from Sigma-Aldrich and used to prepare phosphate buffered saline (PBS, pH of 7.4) solution containing 131 mM NaCl, 5.1 mM Na_2_HPO_4_·2H_2_O, and 1.5 mM KH_2_PO_4_ with highly purified water (18.2 MOhm cm). The PBS solution was degassed before used as receptor and donor solution in the Franz cells.

#### Pig skin as in vitro model

The inside of the outer ear from pig was used as in vitro skin model. Pig ears were purchased from a local abattoir (Strömbäcks Gårdsslakteri, Illstorp, Sweden) and stored at − 80 °C until use. In the case of hydration experiments with NE and HP, the pig ears were gently rinsed, trimmed for hair, and left for 3 h at ambient environment in order equilibrate SC to the ambient relative humidity (39 ± 7%) before initiating the hydration study. To prepare skin membranes to be mounted in the Franz cell for 4E measurements, pig ears were taken out of the freezer and left to thaw at room temperature for about 2 h. The ears were gently rinsed under cold tap water and hair was removed with a trimmer. Next, the ears were sliced in stripes using a scalpel; regions with cartilage were avoided. The skin stripes were dermatomed (Dermatome, Integra LifeSciences, Plainsboro, NJ, USA) to a final thickness of approximately 500 µm. Skin membranes were then punched out (16 mm in diameter) and left on a PBS-wetted filter paper for 3 h at ambient environment in order to achieve approximately the same degree of hydration before storage. Finally, the skin membranes were wrapped in aluminum foil and stored at − 20 °C. Skin membranes were used within one month after preparation.

### Electrical impedance methods

Figure 1 shows a graphical representation of the electrical impedance spectroscopy (EIS) methods used in this work. Note that the Nevisense (NE) and the Hydration Probe (HP) measure the impedance between electrodes in the lateral direction and require direct contact of the electrode with the skin. This is different compared to the 4 electrode (4E) set-up, which measures the impedance across a skin membrane in solution without direct contact between the electrode and the skin membrane. A more detailed description of each method is given below.

#### Nevisense (NE)

The Nevisense (NE) device has been developed as a non-invasive tool for skin cancer diagnosis based on electrical impedance^35,36^. The instrument is equipped with a spring-loaded measuring probe, which controls the pressure of the array of electrodes on the skin measuring site. The measuring probe is disposable and has an area of around 5 × 5 mm^2^. The electrical properties of the skin are probed by application of a harmless electrical current at 35 logarithmically distributed frequencies between 1 kHz and 2.5 MHz, resulting in about 8 s for completion of one measurement. The instrument settings used here were the same as used generally in the clinics with the applied voltage and resulting current limited to 150 mV and 75 µA, respectively, which ensures that test subjects do not experience any uncomfortable sensation^36^.

#### DermaLab hydration probe (HP)

The hydration probe (HP) instrument is a tool that is used in clinical dermatology to evaluate the level of skin hydration. The instrument measures skin conductance at a single frequency equal to 300 kHz, which can be related to the water content of SC on an arbitrary scale. The measuring probe contains an array of 8 pins and is designed specifically to minimize the moisture accumulation due to occlusion of the skin area under the electrode. The probe is equipped with a spring-loaded function, which both controls the applied pressure during the measurement and initiate and stops the measurement. One measurement takes less than 5 s. The hydration software stores up to 8 sequential measurements and automatically calculates the average result provided in µS.

#### Four-electrode Franz cell set-up (4E)

With this method, the electrical impedance spectroscopy (EIS) measurements are performed with a four-electrode setup (4E) employing dermatomed skin membranes mounted in a Franz cell (orifice diameter = 9 mm, V_receptor_ = 6 mL, V_donor_ = 1 mL; PermeGear Inc., Hellertown, USA)^9,37^. The electrodes are connected to a potentiostat from Ivium Technologies (Eindhoven, The Netherlands). The frequency range was chosen between 10 Hz and 1 MHz with 4 frequencies per decade in order to minimize the measurement time to 20 s in total. In order to ensure low current densities in the lower frequency region, the amplitude of the applied voltage was kept between 50–100 mV. The temperature in the Franz cell was controlled by a circulating water system.

### Study design

The hydration experiments were carried out both in vivo on the left forearms of healthy volunteers and in vitro using either intact pig ears in the case of NE and HP measurements or dermatomed skin membranes in the case of 4E measurements. For the NE and HP measurements, the skin site was hydrated with tissues (Salvequick, wound cleanser, Orkla Care AB, Sweden) soaked with a physiological saline solution (150 mM NaCl), while buffered physiological saline (131 mM NaCl) was used in the Franz cell chambers during the 4E measurements. An overview of the study design of the hydration experiments with the three different techniques are presented in Table 1 and detailed in the text below.

**Table 1:** Overview of the study design.

Type	Nevisense (NE) and hydration probe (HP)	4E Franz cell (4E)
In vivo	Four subjects Three regions on left volar forearm Hydration with 150 mM NaCl (aq) Series of 12 time points between 0 and 16 min	Not applicable
In vitro	Four (NE) and three (HP) individual ears Three regions on each pig ear Hydration with 150 mM NaCl (aq) Series of 12 time points between 0 and 16 min	Split-thickness pig skin membranes Hydration in PBS (131 mM NaCl) Six temperatures 12–72 °C Continuous measurements during 3 h

It should be pointed out that the experiments were designed taking into consideration the biological variability between different subjects and between different skin sites. The experimental design minimizes this natural variability as it generates relative changes of the impedance data with the hydration time as the varying parameter. In fact, the standard deviations of the relative change of the impedance data, obtained with the NE and the HP instruments, from different individuals (or ears), were similar as the standard deviations of the corresponding data obtained from different skin regions on the same individual (or ear). By performing systematic two-sided *t*-tests for comparing the mean values of the different data sets, no evidence for any statistically significant differences could be established (see Fig. S1). Based on this conclusion, it is reasonable to treat each hydration series as one individual replicate, irrespective of the origin of the hydration region being either from the same subject (or ear) or from different subjects (or ears).

#### In vivo experiments with Nevisense (NE) and DermaLab hydration probe (HP)

As summarized in Table 1, the in vivo study included 12 hydration sites in total, which were distributed over three different regions on the left volar forearm of four healthy volunteers resulting in 12 separate hydration series (*n* = 12). The area of the hydration region was 3 × 4 cm^2^, which ensured that the measuring probe fitted inside each of the 12 hydration sites by good margin. Each hydration site was marked with a pen; care was taken so that skin areas close to the wrist and the elbow pit were avoided. All in vivo measurements were conducted within two weeks. During this period, the indoor relative humidity was 39 ± 7% and the temperature was 21.3 ± 0.5 °C. The volunteers, who gave informed written consent, included 3 females and 1 male (22–29 years old) and were non-smokers without any known or visible skin diseases. The subjects were asked not to use any skin products and not to wear clothes with long sleeves on the day of measurements. The study was conducted according to the Declaration of Helsinki and approved by the by the Swedish Ethical Review Agency (Dnr 2019-05264).

#### In vitro experiments with Nevisense (NE) and DermaLab Hydration probe (HP)

As summarized in Table 1, the in vitro study included 12 (NE, *n* = 12) or 9 (HP, *n* = 9) individual hydration series in total, which were distributed over four (NE) or three (HP) different ears with three different regions each (3 × 7 cm^2^). A pen was used to mark each hydration site and care was taken to ensure that areas in close proximity to cartilage were excluded.

#### In vitro experiments with four-electrode Franz cell set-up (4E)

The EIS measurement was initiated directly after addition of 1 mL of PBS into the donor chamber in order to cover the initial conditions of the skin membrane. The experiments were performed at room temperature (21.7 ± 0.6 °C) if not stated otherwise. The data was collected according to the following: scans 1–20 were performed with 20 s in between each measurement, scans 21–30 with 1-min intervals, scans 31–40 with 2.5-min intervals, scans 41–50 with 5-min intervals, and finally scans 51–60 with 10-min intervals, which corresponds to a total time of 3 h and 10 min. Moreover, it was decided to perform a more detailed study on the hydration process as a function of temperature to gain a better understanding on how the electrical properties of the skin barrier are affected by these parameters. To achieve this, the same hydration protocol as described above was used at the following temperatures: 12 °C (*n* = 4), 22 °C (*n* = 5), 32 °C (*n* = 8), 42 °C (*n* = 6), 52 °C (*n* = 4) and 72 °C (*n* = 2).

#### Evaluation of the electrical impedance spectroscopy (EIS) results

To enable a complete comparison between the data sets obtained with the different methods, without introducing a wide number of parameters and unnecessary complexity, it was decided to present the EIS data obtained with the NE and 4E methods in three different ways. Here, it should be noted that the HP method only provide one measuring parameter, i.e. the conductance at 300 kHz. To allow for a comparison with this parameter, the conductance measured at a similar frequency with NE and 4E was used.

Next, the fact that both NE and 4E are methods based on impedance spectroscopy allows for a more robust comparison between the data obtained with these methods. Therefore, it was decided to compare two additional parameters from the NE and 4E data with the overall aim to better understand the hydration process. The first parameter was chosen as the total impedance at a low frequency where the SC barrier properties are probed. The second parameter was chosen to be the so-called magnitude index (MIX)^38^, which represents a ratio of impedance magnitudes from one low and one high frequency. To better understand these parameters, it is suitable to briefly explain the fundamental theory of EIS. In brief, the EIS technique measures the total impedance, *Z* (Ohm), which can be can be represented as a complex number consisting of real and imaginary parts. These are defined as *Z* = *Z*_Re_ + *iZ*_Im_, where *Z*_Re_ is the real part and *Z*_Im_ is the imaginary part of the total impedance and *i* is the imaginary number. The impedance data can be presented either as a Nyquist plot where the imaginary part (*y*-axis) is plotted against the real part (*x*-axis) (see Fig. S2), or as a Bode plot, where the magnitude, |*Z*| (Ohm), commonly is plotted on the left *y-*axis and the phase angle, *θ* (°), is plotted on the right *y*-axis; both as a function of the frequency in logarithmic scale (log *x*-axis) (see Fig. S3). The magnitude of the total impedance is determined as |*Z*|= (*Z*_Re_^2^ + *Z*_Im_^2^)^0.5^ and the phase angle is expressed as *θ* = tan^−1^(*Z*_Im_/*Z*_Re_).

Returning to the chosen parameters used to evaluate the present results of the hydration study. The data obtained at 1 kHz are dominated by *Z*_Re_ and are therefore a good approximation of the resistive properties of the skin barrier, which is strongly connected to the permeability properties^9,10^. However, it should be noted that the EIS data in the range between 1 and 10 kHz obtained with the NE technique can be affected by the contact between the electrode and the skin surface, which may be difficult to control. This caveat should be kept in mind for the 1 kHz data obtained with the NE. Still, it was decided to use this parameter due to the fact that it can be straightforwardly compared with the data obtained with the 4E methodology, where the 1 kHz data represent the skin membrane resistance in a consistent and well established manner^9,39,40^. Further, previous measurements performed with a noninvasive Nevisense probe showed that the NE impedance data can be expressed as the ratio of the impedance magnitudes at two particular frequencies of 20 and 500 kHz, which greatly reduces the complexity of the experimental data^38^. This ratio is referred to as the MIX index and defined as MIX =|*Z*_20kHz_|/|*Z*_500kHz_|^38^. In particular, the MIX index reflects changes of both resistive properties (i.e. SC barrier properties) and capacitive currents related to the dielectric properties of SC (e.g. extracellular lipid lamellae and keratin properties). To enable a comparison with the data obtained with the 4E methodology, it was decided to utilize a similar MIX ratio. However, due to the fact that the two instruments (i.e., NE versus 4E) are different, and also that the skin is exposed to different environments during these measurements, it is not possible to use the impedance magnitudes from the same frequencies of 20 kHz and 500 kHz. Instead, the selection of the MIX frequencies, which were decided to be 1 kHz and 10 kHz in the case of 4E measurements, was based on the qualitative relationship between the impedance magnitude and phase shift (see Fig. S3). In other words, the 4E data on impedance magnitude and phase shift are similar to the NE data obtained at 20 kHz and 500 kHz, but at significantly lower frequencies of 1 kHz and 10 kHz, respectively.

## Results

**Figure 2:**
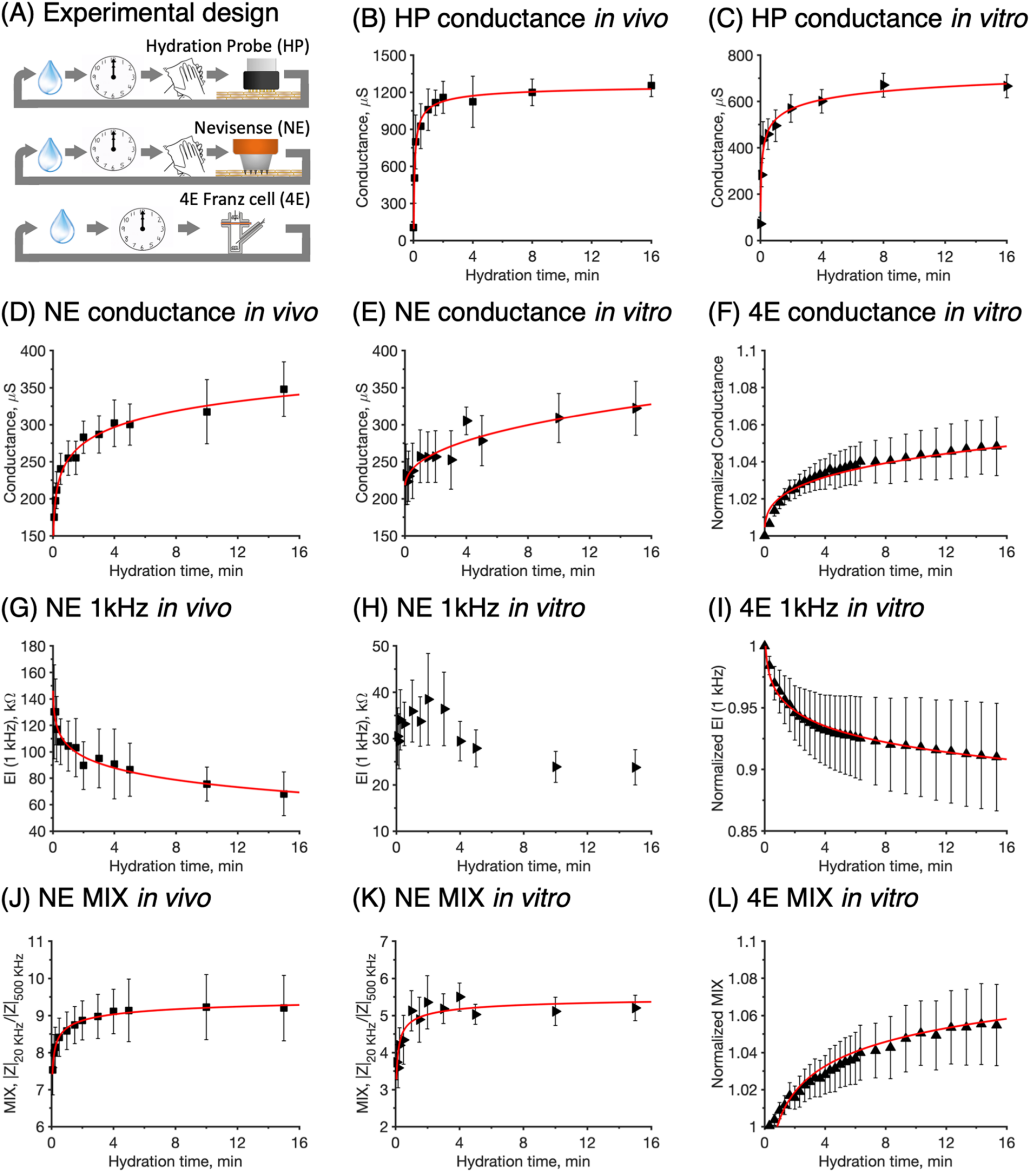
(**A**) Study design to investigate the kinetics of skin hydration with the Hydration Probe (HP), Nevisense (NE), and four-electrode impedance spectroscopy (4E). The effect of hydration on skin conductance measured with (**B**) HP in vivo and (**C**) HP in vitro, (**D**) NE in vivo (at 315 kHz), (**E**) NE in vitro (at 315 kHz), and (**F**) 4E in vitro (at 251 kHz). The effect of hydration on the impedance at 1 kHz measured with (**G**) NE in vivo, (**H**) NE in vitro, and (**I**) 4E in vitro. Finally, the effect of hydration on the MIX parameter measured with (**J**) NE in vivo, (**K**) NE in vitro, and (**L**) 4E in vitro. Error bars represent ± SEM where *n* = *12* for HP in vivo (**B**) and *n* = 9 for HP in vitro (**C**), *n* = *12* for NE in vivo (**D**,**G**,**J**) and NE in vitro (**E**,**H**,**K**); 4E: *n* = 5 (**F**,**I**,**L**).

**Figure 3:**
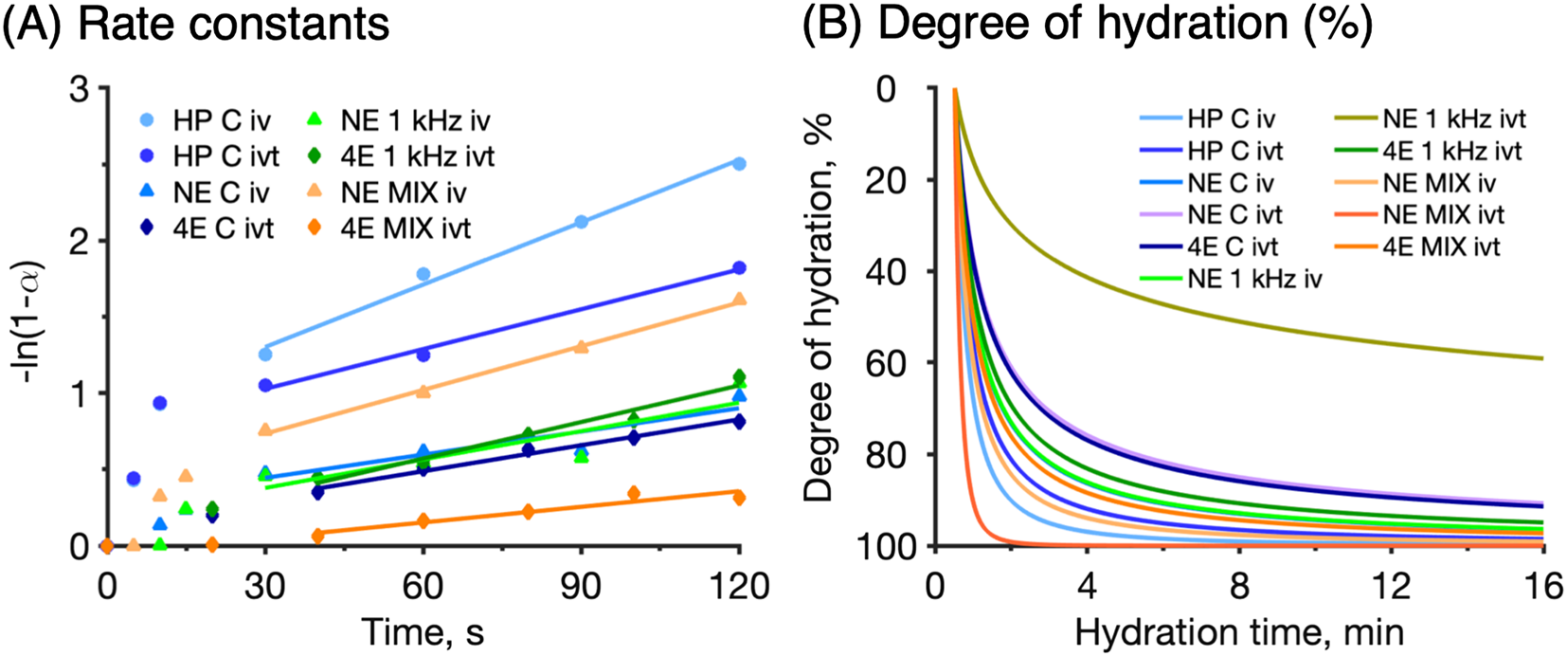
Modelling of experimental data. (**A**) First order decelerating kinetics rate constants determined between 30 and 120 s according to Eq. (2). Rate constants and R^2^ values are summarized in Table 2. (**B**) Degree of hydration calculated according to Eq. (3).

**Figure 4:**
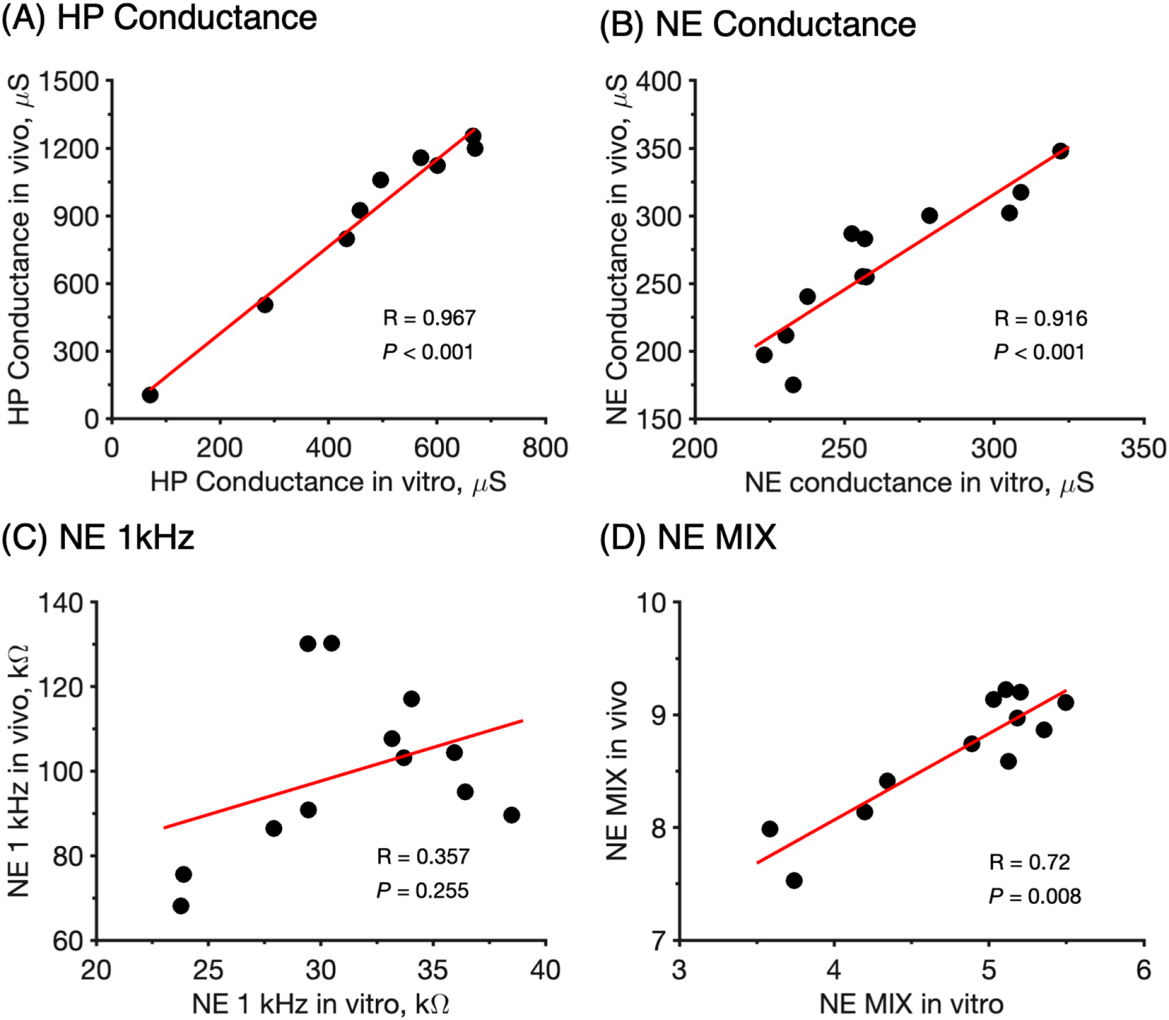
Spearman correlation patterns between in vivo–in vitro data of (**A**) HP conductance, (**B**) NE conductance, (**C**) NE 1 kHz, (**D**) NE MIX parameter. Data are shown as mean values (*n* = 12 and *n* = 9 for HP conductance in vivo and in vitro, respectively; *n* = 12 for NE in vivo and in vitro).

**Figure 5:**
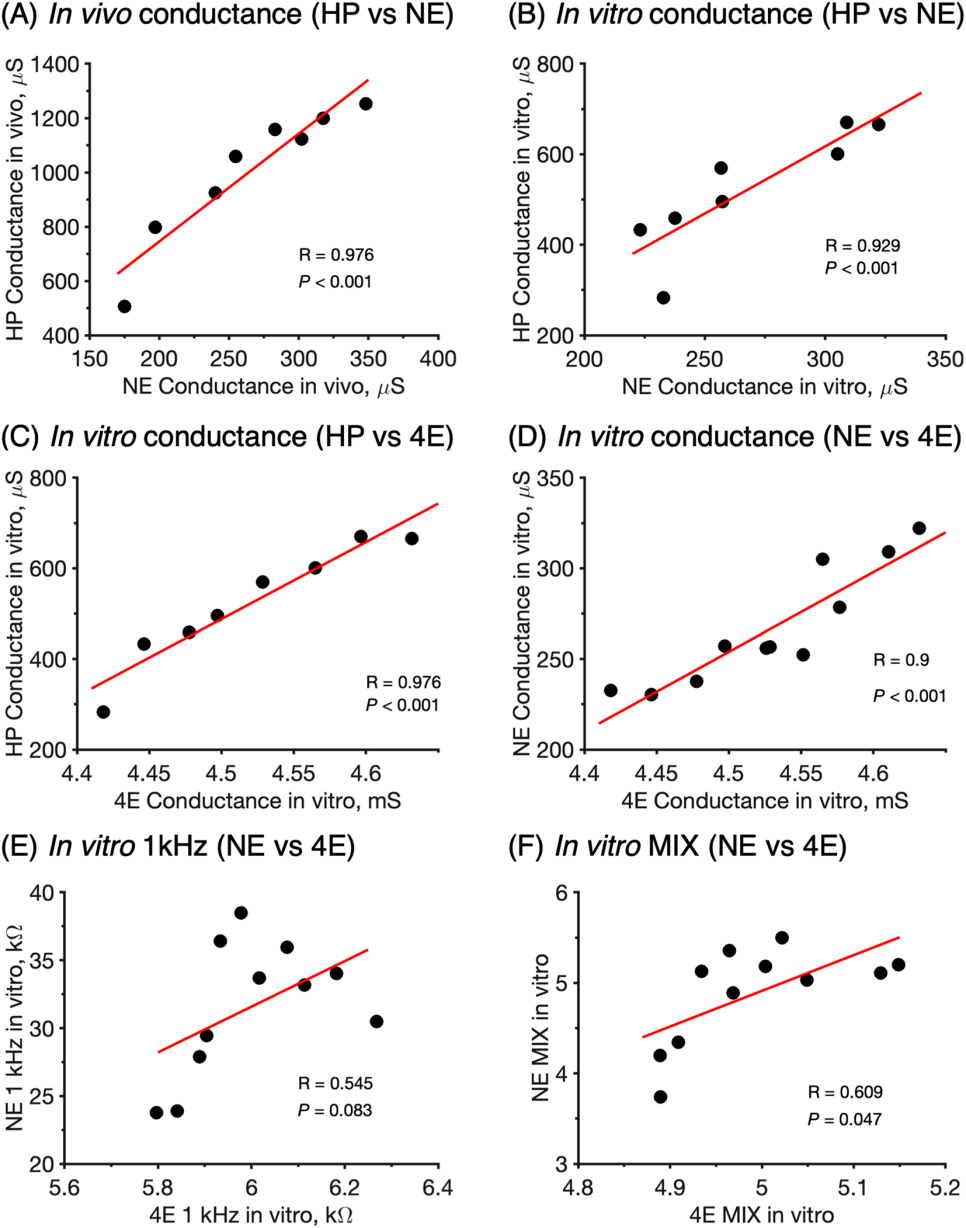
Spearman correlation between different methods. Note that the data are either from in vivo or in vitro measurements. (**A**) In vivo conductance. (**B**–**D**) In vitro conductance. (**E**) In vitro 1 kHz. (**F**) In vitro MIX. Data are shown as mean values (HP: *n* = 12 and *n* = 9 for in vivo and in vitro*,* respectively; NE:* n* = 12 for in vivo and in vitro; 4E: *n* = 5).

**Figure 6:**
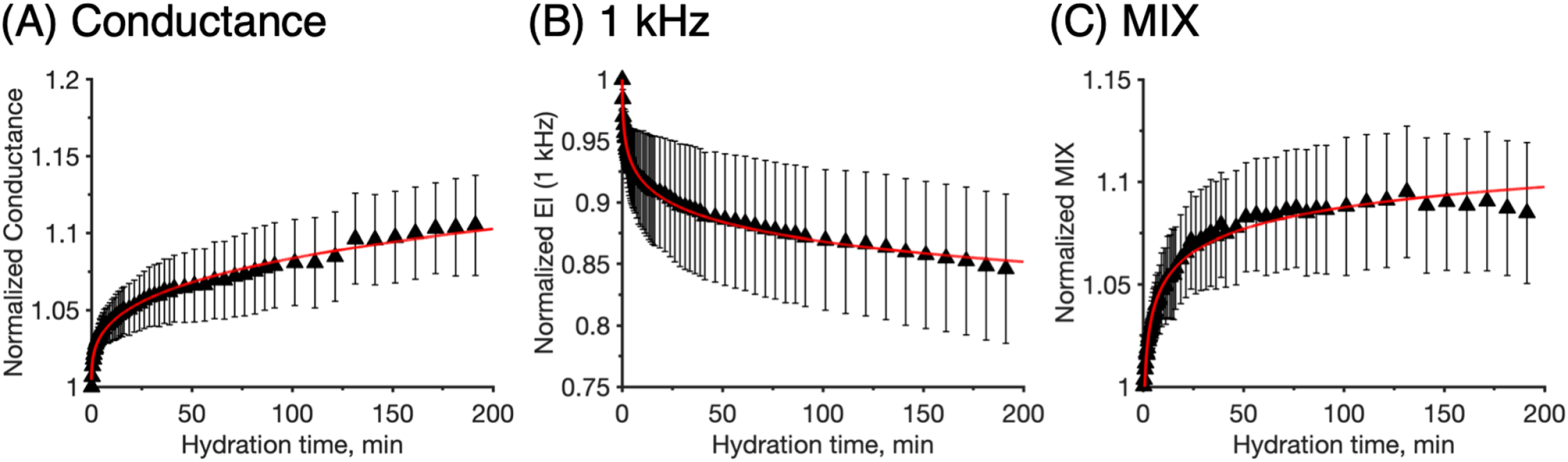
Normalized electrical impedance of skin measured with the four electrode Franz cell setup (4E) during 180 min of hydration exposure. (**A**) conductance at 251 kHz, (**B**) 1 kHz resistance, (**C**) MIX parameter. Experimental data is fitted with a power law. Error bars represent ± SEM (n = 5).

**Figure 7:**
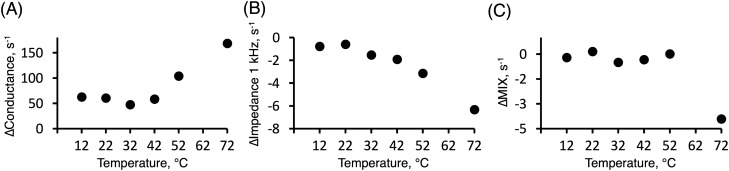
The rate of change, ∆ (s^−1^), determined from the slope of linear fits between 60 and 150 min of hydration experiments performed at different temperatures. These experiments were performed in a similar manner as compared to the results presented in Fig. 6 for 22 °C. (**A**) Conductance data at 251 kHz. (**B**) 1 kHz resistance data. (**C**) MIX parameter.

A general aim of this study was to investigate the dynamics skin hydration by means of different impedance-based methods. For this purpose, three techniques were used to measure changes of the electrical properties of the SC during exposure to the same hydration protocol both in vivo and in vitro. The results are structured according to the following. First, we focus on the effect of hydration on the impedance data obtained from the different methods (Fig. 2). Next, we focus on the time required to reach an initial stable signal, which can be interpreted as close to full skin hydration (Fig. 3 and Table 2). Subsequently, we analyze correlation patterns between the in vivo and in vitro data (Fig. 4) and between data obtained from the different methods (Fig. 5). Finally, the effect of prolonged hydration at different temperatures are investigated (Figs. 6, 7, and Table 3).

**Table 2:** Summary of hydration dynamics of the initial hydration process according to the first order kinetics model.

Method	First order kinetics (Fig. 3A)			Degree of hydration, ${\theta }_{h}$, (Fig. 3B) at time ${t}_{h}$
	$k$	Hydration time,${t}_{h}$	R^2^	
HP conductance in vivo	0.0136	4	0.991	97%
HP conductance in vitro	0.0087	6	0.993	95%
NE conductance in vivo	0.0051	10	0.831	96%
NE conductance in vitro	–	–	–	–
NE MIX in vivo	0.0095	5	0.997	91%
NE MIX in vitro	–	–	–	–
NE 1 kHz in vivo	0.0062	8	0.764	93%
NE 1 kHz in vitro	–	–	–	–
4E conductance in vitro	0.0049	10	0.963	88%
4E MIX in vitro	0.0034	15	0.904	97%
4E 1 kHz in vitro	0.0080	6	0.964	88%

First order rate constants, $k$ (s^−1^), obtained by linear regression (see Fig. 3A) and estimated hydration times, ${t}_{h}$, (in minutes) required to reach 95% conversion, i.e. $\alpha$ = 0.95 in Eq. (2). The degree of hydration, ${{\varvec{\theta}}}_{{\varvec{h}}}$, was calculated with Eq. (3) (see Fig. 3B) together with the hydration time,${t}_{h}$. If the degree of hydration is close to 95%, it supports the first order kinetics model with 95% conversion.

**Table 3:** Summary of hydration dynamics of the initial hydration process as a function of temperature according to the first order kinetics model.

T (°C)	Conductance			1 kHz			MIX		
	$k$	Time	R^2^	$k$	Time	R^2^	$k$	Time	R^2^
12	0.0057	9	0.985	0.0085	6	0.998	–	–	–
22	0.0048	11	0.962	0.0080	6	0.964	0.0034	15	0.904
32	0.0054	9	0.996	0.0072	7	0.990	0.0027	18	0.836
42	0.0044	12	0.993	0.0074	7	0.999	0.0033	15*	0.997
52	0.0034	15	0.946	–	–	–	0.0026	19*	0.903
72	0.0046	11	0.978	–	–	–	0.0025	20*	0.984

The calculated parameters are based on impedance measurements with the 4E setup according to Eqs. (1) and (2) using input data of 4E conductance at 251 kHz, 4E resistance at 1 kHz, and 4E MIX index based on impedance at 1 kHz and 10 kHz. In these calculations, the $\alpha$ values were obtained between 0 and 16 min. However, times marked with * indicate that $\alpha$ were obtained between 0 and 6 min, due to deviating behavior at longer times.

### The effect of hydration on the electrical impedance properties of skin

The experimental results from the hydration study are presented in Fig. 2 together with fitted curves based on a power law function ($Response=a{b}^{t}+c$, see red solid curves in Fig. 2). Note that the impedance data from the in vitro 4E measurements (Fig. 2F,I, and L) are normalized due the substantial biological variation of the electrical impedance properties between different excised skin membranes, which is commonly observed in similar Franz cell in vitro measurements^9,33,41^. The three employed techniques are different and measures slightly different electrical properties of the skin. However, it is possible to compare the results in the following manner.

Firstly, the results from the conductance measurements with the HP probe, operating at a single frequency of 300 kHz, are presented in Fig. 2B (in vivo) and C (in vitro). These results can be compared with the corresponding conductance data obtained at the closest frequency with both the NE method (i.e., conductance = 1/|*Z*_315kHz_|) in Fig. 2D (in vivo) and E (in vitro), and the 4E technique (i.e., conductance = 1/|*Z*_251kHz_|) in Fig. 2F (in vitro). Secondly, Fig. 2 G–I show the impedance data obtained at 1 kHz from NE in vivo, NE in vitro, and 4E in vitro, respectively. Finally, the MIX index from NE in vivo, NE in vitro, and 4E in vitro are presented in Fig. 2 J–L, respectively.

Taken together, the results in Fig. 2 show good agreement in general, both between results obtained with the same method in vivo or in vitro and when comparing the same parameter obtained with different methods. However, a closer evaluation of the results reveals several differences, which are detailed in the following.

Starting with the conductance data presented in Fig. 2 B–F; the results show that the various data sets are comparable. However, the drastic increase of the conductance, which normally is associated with an increase of the water content of the SC^9,32,33^, occurs at slightly different times and the profiles of the curves are different. Qualitatively, the change occurs fastest for the results obtained with the HP probe in vivo (Fig. 2B), while the in vitro data from the same instrument seem to level off after a slightly longer time (Fig. 2C). Further, the curves in Fig. 2B and C show similar shapes with a rapid onset followed by a stable plateau after about 3–4 min. It can also be noted that the absolute values are approximately two times higher in the case of the in vivo measurements on healthy human subjects as compared to the in vitro measurements, which were obtained with full-thickness pig ears. Next, by comparing the HP conductance results with the corresponding data from the NE and 4E methods, it is clear that the hydration induced change of the conductance is less rapid in the latter cases. In particular for the data set obtained with the 4E method in vitro, which also appears to follow a somewhat different signal response profile with no stable plateau.

Turning to the impedance data at 1 kHz (Fig. 2 G–I), which mainly depend on the resistance of electrical charge transfer across the SC barrier, it is clear that also this parameter decreases due to the hydration process. In general, the results show that hydration results in a decrease of the skin membrane electrical resistance, which is in line with previous investigations^9,11^. Strikingly, the impedance data at 1 kHz have a similar, but inverted, appearance as compared to the corresponding conductance data obtained with the same instrument, although the measuring frequency is different (i.e., 315 kHz for NE and 251 kHz for 4E). In other words, the 1 kHz response curves (Fig. 2 G–I) have an initial region where the skin barrier electrical resistance decreases relatively rapid (0–2 min), followed by a region where the resistance decreases in a less prominent manner. Importantly, no stable region is observed, except perhaps for the data from the NE in vitro measurement (Fig. 2H). However, the NE in vitro data were in general associated with large variability. This is most likely due to superficial cartilage ridges of the inner pig ear, which may influence the electrical impedance currents between the microneedle electrodes of the NE probe, leading to noise and unwanted variability. The reason why this is not causing disturbances in the measurements with the HP probe is probably that this electrode is flat, as compared to the microneedle electrodes of the NE probe that penetrates superficially into the SC layer. Here, it can be noted that there is an optional probe for the NE instrument with flat electrodes, which may generate in vitro impedance data from measurements on intact pig ears with less noise, similar to the in vitro data obtained with the HP probe that operates with flat electrodes (Fig. 2C). Alternatively, it is likely that NE measurements performed in vitro with the microneedle electrode probe on skin sites without any superficial cartilage in the underlying tissue, such as the abdominal skin regions, would generate impedance data with less variability. These issues were, however, not investigated in further detail in the present work where all in vitro NE measurements were performed with the microneedle electrode probe on skin from the inside of pig ears.

Finally, the MIX parameter (Fig. 2 J–L) is in all cases observed to increase as a response to continuous skin hydration. This observation can be explained by the fact that the impedance at low frequencies (20 kHz for NE or 1 kHz for 4E) is relatively less attenuated, as compared to the impedance at high frequencies (500 kHz for NE or 10 kHz for 4E), upon exposure to skin hydration. In other words, the numerator of the MIX ratio (low frequency impedance) decreases less as compared to the denominator (high frequency impedance), thereby resulting in an increase of the MIX ratio. Still, it is relevant to point out that in general terms, hydration leads to a decrease of the electrical impedance at all frequencies. On a more biophysical note, the observed increase of the MIX parameter implies that hydration has a relatively stronger impact on the capacitive currents (high frequency impedance) inside the SC, as compared to the resistive currents (low frequency impedance). A closer evaluation of the MIX indices, allows us to conclude that there are some differences between the different methods. In particular, the changes of the MIX index measured by the NE method, both in vivo and in vitro (Fig. 2J,K), show a strong increase during the first 2 min, after which the curves level out and reaches stable plateau values. This should be compared to the MIX results obtained with 4E method (Fig. 2L), which show a less pronounced initial response and no clear signs of levelling out at a stable plateau.

Taken together, the results in Fig. 2 show that all impedance techniques can be used to successfully investigate the effect of hydration on the electrical properties of skin both in vivo and in vitro. Nevertheless, it can be seen that not all response curves have reached a stable reading during the 16 min of experimental time that was used as hydration time; in particular the measurements performed with the NE and 4E methods. This implies that the hydration process has not reached a steady state and that longer exposure times for hydration is required to achieve a fully hydrated skin membrane.

### Hydration times and the degree of hydration

The physicochemical properties of the skin barrier at full hydration are expected to favor high transport rates for transdermal delivery and extraction of low molecular weight biomarkers^10,13^. Therefore, knowledge of the time required to hydrate the skin and transform it into a highly permeable or readily extractable tissue is of great value. To enable a more detailed evaluation of the hydration rate, with the aim of estimating the time required to reach a close to fully hydrated skin tissue, we reevaluated the data in Fig. 2 according to a simple kinetics model. Since the skin impedance data in Fig. 2 in general are associated with a decelerating behavior upon continuous exposure towards hydration, it is suitable to model the experimental data according to first order deceleration kinetics. Accordingly, the process can be described as conversion of SC from an initial state into a more hydrated state; i.e., ${SC}_{\mathrm{initial}}+{H}_{2}O\to {SC}_{\mathrm{hydrated}}$. The extent of the hydration process can be modelled by introducing a conversion factor $\alpha$, which is defined according to Eq. (1) where $EIR$ is the electrical impedance response at time point $t$, while the indices $i$ and $f$ refers to the initial ($t$ = 0) and final ($t$ = 16 min) measurements, respectively.

1 $$\alpha = \frac{{EIR_{i} - EIR_{t} }}{{EIR_{i} - EIR_{f} }}$$

The value of $\alpha$ ranges between zero and unity and for a first order reaction the following equation is valid where $k$ is the rate constant and $t$ is the reaction time:

2 $$- \ln \left( {1 - \alpha } \right) = kt_{h}$$

Equation (2) can be used to determine the rate constant $k$ by plotting $-\mathrm{ln}\left(1-\alpha \right)$ as a function of the hydration time ${t}_{h}$, which is expected to yield a straight line with the slope equal to the rate constant $k$. The rate constants were determined from linear regression analysis of the experimental data between 30 and 120 s according to Eq. (2). The reason for limiting the regression analysis between these time points is that the data obtained before 30 s were associated with large variations, while the data obtained after 30 s in most cases were linear. However, in a few cases, also the data after 120 s deviated from a linear behavior. These observations limited the time interval where all measurements could be evaluated within the same time interval. Thus, for consistency, a limited time interval between 30 and 120 s was used. The results from the regression analysis are presented in Fig. 3A and compiled in Table 2.

Based on the rate constants ($k$) in Table 2, together with Eq. (2), it is possible to calculate the time (${t}_{h}$) required to reach a certain hydration degree. Here, it was decided to determine the time required to reach 95% hydration by employing Eq. (2) with $\alpha$ = 0.95 (i.e. 95% of conversion). The results from these calculations are summarized in Table 2. Note that the limit of conversion (i.e., $\alpha$ = 0.95) represents an arbitrarily selected limit to enable a comparison between the different data sets.

As illustrated in Fig. 3A and Table 2, the first order decelerating kinetics model is valid for the experimental data in most cases with coefficients of determination (R^2^) above 0.75. Exceptions to this are the results obtained with the NE method in vitro, which were excluded in Fig. 3A and Table 2 due to poor fitting. As mentioned above, the deviating results from the in vitro measurements with NE are likely due to disturbances originating from superficial cartilage regions of the inside of the pig ear, leading to unsatisfactory impedance data.

To validate the calculated hydration times in Table 2, it was decided to estimate the degree of hydration after these times with an alternative approach. This was achieved by taking the time derivative of the electrical impedance response according to Eq. (3) below, where ${\theta }_{h}$ represents the degree of hydration.

3 $$\theta_{h} = { }\frac{\Delta EIR}{{\Delta t}} = \frac{{EIR_{n} - EIR_{n - 1} }}{\Delta t}$$

In Eq. (3), $\Delta EIR$ represents the change of the impedance response (obtained from the power law fittings of the experimental data; see the red curves in Fig. 2), $n$ is the number of the data point (1, 2, 3 … $n$), and $\Delta t$ is time interval between two data points (set to 1 s).

As previously demonstrated (Fig. 2), even a short hydration exposure of the skin results in a drastic change in measured response. In particular, for the HP in vivo measurements, the skin conductance increased 6 times during the initial 30 s of hydration (Fig. 2B). It is not unlikely that this drastic change is due to surface hydration leading to enhanced contact between the electrode and the skin. Potentially, this mechanism may be relevant both for the HP and the NE probes, while the fact that there is no contact between the skin and the electrodes of the 4E setup could explain the absence of such a drastic change in this case (Fig. 2). In order to reduce any bias from this effect, it was decided to exclude data obtained within the first 30 s in the power law fitting analysis. Moreover, due to the fact that the different instruments generate significantly different absolute values (Fig. 2), it was decided to normalize the data in order to compare the results from the different techniques. This normalization was done with respect to the initial values, starting from 30 s. Finally, the changes of the degree of hydration were calculated according to Eq. (3) and plotted in Fig. 3B. As shown in Fig. 3B, starting from an initial (non-hydrated) state, the degree of hydration is rapidly approaching high degrees within the first minutes, which is in line with Fig. 2. For example, already within minutes the degree of hydration is above 80% of the initial value in most cases. It should be noted, however, that the initial degree of hydration likely reflects a normal hydration state and not completely dry skin. To compare the data sets in a more systematic manner, the degree of hydration at the hydration times calculated from the kinetic model (Table 2) were estimated from the curves in Fig. 3B. The calculated values are summarized in Table 2.

In general, when R^2^ is above 0.95 in Table 2, the estimated hydration time is 6 ± 2 min (average ± SD), while the average degree of hydration is 92 ± 4%. This can be compared to the average hydration time, taking all data into account, which is 8 ± 4 min while the corresponding average degree of hydration is 93 ± 4%. On the other hand, the estimated hydration times varies somewhat depending on the method used. For example, the estimated average hydration times, taking into account both in vivo and in vitro results, for HP, NE, and 4E are 5 min, 8 ± 3 min, and 10 ± 5 min, respectively. The corresponding average degrees of hydration after these exposure times are 96, 93 ± 3%, and 91 ± 5%, respectively. Taken together, this evaluation implies that the estimated hydration times and degrees of hydration are in relatively good agreement overall. To conclude, the results in Fig. 3 and Table 2 indicate that the hydration process, corresponding to 95% conversion of the measured electrical impedance signal [i.e., $\alpha$ = 0.95 in Eq. (2)], occurs within about 8 ± 4 min overall. This conclusion is in line with average degree of hydration (93 ± 4%), which was calculated with Eq. (3) in combination with the estimated hydration times from the first order kinetics model.

### Correlation patterns between in vivo–in vitro data and between different electrical impedance methods

To facilitate research and development related to skin in general, it is important to have safe, reliable, and uncomplicated in vitro tools that mimic the in vivo situation with high precision and accuracy. Furthermore, it is safe to assume that different institutions, both within academia and in industry, are equipped with different types of measuring instruments. Therefore, there is a great need to establish in vivo*–*in vitro correlations, as well as correlations between different techniques employed either in vivo or in vitro. To approach this issue, we correlated the in vivo results with the in vitro results obtained with HP, NE and 4E using the Spearman correlation test (note that 4E is an in vitro methodology only). All experiments were designed to facilitate this kind of correlation analysis by, as far as possible, employing identical hydration protocols. The results of in vivo*–*in vitro correlations are presented in Fig. 4. In a similar manner, Spearman correlation tests were performed to compare the results obtained by the different techniques; the cross-method correlations are presented in Fig. 5.

The results in Fig. 4 show that there is very strong correlation between the in vivo and in vitro data in all cases, except for the 1 kHz data obtained with the NE method. Again, the reason for this is most likely due to superficial cartilage residing in the inside of the pig ears that interferes with the impedance current between the microneedle electrodes of the NE probe in the case of the present in vitro NE measurements.

Figure 5 shows overall good cross-method correlations for the conductance data obtained either in vivo or in vitro with the HP, NE, and 4E methods (Fig. 5 A–D). This observation is encouraging as it implies that several techniques can be used to investigate the process of skin hydration. Less satisfactory correlations are observed for the in vitro 1 kHz data and the relatively more complex MIX ratio (Fig. 5E,F). Most likely, these unsatisfactory correlations can be associated primarily to the impedance data obtained by the in vitro NE measurements due to presence of interfering superficial cartilage of the pig ears, while the impedance data obtained by the 4E method are seemingly satisfactory.

### The effect of prolonged hydration and temperature on the electrical properties of skin

As shown in Fig. 2, stable and constant values of the electrical skin properties were obtained with the HP probe within 16 min of skin hydration (Fig. 2B,C). In contrast, the corresponding results obtained with the NE and 4E methods indicated further changes of the impedance response; in particular in the case of the 4E data. Therefore, it was decided to perform prolonged hydration experiments using the in vitro 4E setup to investigate if steady-state conditions, in terms of stable electrical impedance properties of the skin membrane, could be achieved.

#### Prolonged hydration studied with the 4E methodology

The effect of prolonged hydration was evaluated over 3 h in a similar manner as described above (Fig. 2F,I, and L). The results are presented in Fig. 6. As aforementioned, it is well-known that in vitro electrical impedance data from excised skin membranes are associated with substantial biological variation^9,33,41^. For this reason, the present 4E impedance data are normalized (Fig. 6). Still, the results in Fig. 6 show relatively large deviations despite this normalization, which implies that the relative change of the impedance data due to hydration is also prone to natural biological variation. Alternatively, it is likely that the initial hydration states of the skin membranes were not identical, which could introduce a significant source of variation of the initial hydration response. In support of the latter interpretation is the observation that the variations (i.e., the ± SEM in Fig. 6) propagate primarily during the initial time region and then remain more or less constant. The qualitative response pattern of the impedance data, due to prolonged skin hydration, is however similar in all cases with two different stages with apparently different kinetics (Fig. 6).

In the initial process, the conductance (Fig. 6A), the resistance (Fig. 6B), and the MIX parameter (Fig. 6C), are changing abruptly. This initial stage is followed by a second process where the changes of the impedance data proceed at a slower rate and, seemingly, without any stable endpoint (Fig. 6A,B). Turning to the MIX parameter (Fig. 6C), as concluded in relation to Fig. 2L, this parameter increases as a response to hydration, but the response is moderate and do not reach a stable value after 16 min of hydration exposure (Fig. 2L). However, as shown in Fig. 6C, the MIX parameter reaches a stable and relatively constant value after approximately 60 min. This can be explained by that the rates of change of the low frequency impedance (numerator of the MIX ratio) and high frequency impedance (denominator of the MIX ratio) balance each other, thereby resulting in a constant MIX ratio. In other words, the steady state MIX plateau indicates that the skin membrane capacitance increases at the same rate as the skin membrane resistance decreases.

Taken together, two important points should be highlighted with respect to the results in Fig. 6. First, the results confirm that the hydration process occurs in two stages and that the most drastic change of the impedance signal is observed already within a few minutes. This implies that our evaluation based on impedance data within the first 16 min of hydration (Figs. 2 and 3) is valid for the initial hydration process only, which is important to keep in mind. Secondly, the subsequent slower hydration process does not follow the same kinetics as the faster initial hydration process, which follows first order decelerating kinetics. Instead, there is a clear linear trend in the changes observed after about 60 min (Fig. 6). This observation implies that the rate of the second hydration process can be qualitatively evaluated by analyzing the slope of the linear change. To investigate this issue in more detail, it was decided to perform similar hydration experiments as presented in Fig. 6, but at different temperatures, with the aim to determine the kinetics of the initial and second hydration processes as a function of temperature.

### The effect of temperature on the hydration kinetics

The impedance measurements with the different methods presented above were performed at one fixed temperature (i.e. room temperature) to enable a thorough investigation of the hydration process. Nevertheless, previous investigations have illustrated that both hydration and temperature strongly influence the physicochemical properties of the SC barrier, which is expected to greatly affect the electrical impedance properties of the skin barrier^9,42–44^. These previous studies are, however, mainly focused on the equilibrium properties of the skin barrier at specific temperatures and water contents and not on the dynamics of the hydration process. Therefore, the aim here was to investigate the dynamical aspects of the initial and secondary hydration processes at different temperatures to provide a deeper understanding of the hydration process. Therefore, it was decided to investigate the hydration process in vitro by performing similar 4E impedance experiments as presented in Fig. 6 at 12 °C, 22 °C, 32 °C, 42 °C, and 52 °C. In this temperature range, no clear thermotropic phase transitions are expected based on previous DSC (differential scanning calorimetry) studies on pig SC^45^. In addition, it was decided to include one temperature above the thermotropic lipid melting region, which occurs between 60 and 70 °C for hydrated pig SC^45^. For this reason, experiments were also performed at 72 °C.

To evaluate the dynamics of the initial hydration process, the impedance data were analyzed according to the first order decelerating kinetics model, following the same approach as described above (see Fig. 3A and Table 2). The results from this analysis are summarized in Table 3. In general, the regression analysis showed satisfactory values of the coefficients of determination (R^2^ > 0.84 in all cases).

The results in Table 3 show that the estimated hydration times for the initial process are similar, irrespective of temperature between 12 and 72 °C. However, the values vary slightly depending on which impedance data that was used to derive the hydration times. For example, the fastest hydration time were obtained from the 1 kHz data (7 ± 1 min, average ± SD), followed by the conductance data (11 ± 2 min), while the longest time were obtained from the MIX data (17 ± 2 min). In addition, it should be pointed out that the 1 kHz data obtained at 52 °C and 72 °C, and the MIX data obtained at 12 °C, deviated too much to allow for a solid regression analysis, which is the reason for omitting the results from these temperatures in Table 3.

In contrast to the initial hydration process, which is more or less independent of the temperature, the subsequent hydration process showed an interesting temperature dependance. To evaluate the second hydration process, the slopes of the linear change of the impedance data between 60 and 150 min were analyzed with respect to temperature; the results are presented in Fig. 7.

In Fig. 7, the impedance data are presented in terms of the rate of change, ∆ (s^−1^), of the linear regime between 60 and 150 min, of the conductance (Fig. 7A), impedance at 1 kHz (Fig. 7B), and the MIX parameter (Fig. 7C). Notably, an increasing temperature results in clear effects on all impedance parameters, but the response pattern varies. Starting with the conductance data (Fig. 7A); a constant rate of increase, ∆ (s^−1^), is observed between 12 °C and 42 °C, while the rate is increased at 52 °C and significantly so at 72 °C. The latter observations imply that charge carriers inside the skin barrier become increasingly more unrestricted to respond to the alternating current operating at 251 kHz above about 42 °C.

Next, the rate of decrease of the skin resistance, i.e., negative ∆ (s^−1^) values of the impedance at 1 kHz, is observed to be constant at 12 and 22 °C. However, at elevated temperatures the rate of decrease is growing in a more or less linear manner. This linear decrease implies that the skin membrane resistance, which is connected to charge transfer over longer distances (i.e. the frequency of the alternating current is 1 kHz), becomes less and less impeded.

Finally, the MIX parameter is observed to be constant between 12 and 52 °C, which means that ∆ (s^−1^) is close to zero (see Fig. 6C). As concluded above in relation to Fig. 2, this can be explained by that the rates of change of the impedance data from low and high frequencies balance each other to yield constant MIX ratios. In other words, the skin membrane capacitance increases at more or less the same rate as the resistance decreases, irrespective of the temperature. However, a distinct decrease in the MIX parameter is observed at 72 °C indicating that the change of the low frequency impedance decreases significantly more, in relative terms, as compared to the high frequency impedance.

## Discussion

This work presents a detailed investigation of how skin hydration influences the electrical impedance properties of the skin barrier in vivo and in vitro. Methods based on electrical impedance are well-established in dermatology and cosmetology and commonly applied to evaluate skin hydration at steady-state conditions^32^. However, the dynamics of the hydration process remain less studied. Further, there is a clear need for establishing correlation patterns between the in vivo and in vitro situation as well as between results obtained by different impedance-based methods. These problems were addressed in the present work by employing the Hydration Probe (HP), the Nevisense technique (NE), and a four-electrode setup mounted in a Franz cell (4E), in combination with a well-defined hydration protocol.

### Short time hydration is suggested to mainly result in filling of SC voids

In general, the results in Fig. 2 clearly illustrate that the skin barrier electrical impedance is greatly affected by the hydration process and that the change of the electrical response can be divided into two stages; a rapid initial response followed by a second slower response. From a detailed kinetics analysis of the impedance results from the 16 min hydration protocol (Fig. 3A), the initial hydration process was concluded to occur within 8 ± 4 min in average (Table 2). This value corresponds to 95% conversion of the measured electrical impedance signal, i.e., $\alpha$ = 0.95 in Eq. (2). This conclusion is supported by the theoretical estimations of the degree of hydration (Fig. 3B), which were based on power law fitting of the impedance responses and calculated according to Eq. (3), resulting in an average degree of hydration of 93 ± 4 including all data.

To understand these observations, a relevant starting point is to recapitulate the characteristic impedance behavior of the SC. To a first approximation, the SC impedance properties can be modelled as a parallel arrangement of a resistor and a capacitor^9,34,41–44^. The resistor is associated with ion conductive pathways in SC, which may include the extracellular and intracellular routes, as well as currents through the shunts^9,34,41–44^. The capacitor is associated with restriction of ion transfer at low conductive lipid and lipid-protein domains of the SC^9,34,41–44^. The relevant question here is, how are these features connected to the impedance results observed during the initial short-term and the second long-term hydration processes.

Starting with the initial hydration processes, it seems unlikely that this short hydration time (8 ± 4 min in average) would result in any significant structural rearrangements of the SC extracellular lipid lamellae, or allow for water transport into the corneocytes to induce major swelling, as these processes are expected to occur over significantly longer time scales. Similarly, the fact that the HP conductance remains constant after about 4–6 min (Fig. 2) implies that the conductance pathways are unaffected by any hydration induced changes of the biophysical properties of the SC, which are expected to occur after longer times. Considering these findings, it is more likely that the initial hydration process is related to preexisting air-filled voids of the most superficial layers of SC, such as hair follicles, sweat glands, and furrows, that become filled with aqueous saline solution during the initial hydration treatment. This would lead to formation of highly conductive pathways for charge transfer in the superficial regions of the electrical current between the HP electrodes. Further, low skin conductance is expected if this route is not connected with charge carriers in a continuous manner, going from one electrode, into the SC, and finally back to another electrode (see Fig. 1). In fact, the lowest conductance is observed for the initial data points, implying that air-filled voids are preexisting, which is not unlikely considering that the superficial SC layers are in direct contact with the relatively dry outer milieu. Taken together, the fact that a high conductance pathway is established rapidly (within a few minutes) implies that electrical currents are allowed to pass via an easily accessible and superficial pathway residing in the uppermost skin barrier.

The same line of reasoning can be applied to the results obtained with the NE method (Fig. 2). However, based on the data obtained with the NE method the hydration time was concluded to be slightly longer (i.e., 8 ± 3 min, Table 2). A likely explanation for the difference between the HP and NE results is that the HP instrument has flat electrodes that primarily probe the superficial regions of the SC. In contrast, the microneedle electrode array of the NE instrument is slightly submerged into the SC tissue and therefore expected to probe deeper voids of the SC tissue. In other words, once the superficial voids are filled with electrolyte solution, the HP probe measures high conductance, while the electrical current between the deeper NE electrodes still encounters unfilled voids. Effectively, this is expected to result in a slightly longer hydration time. A similar assertion may also explain why the estimated hydration time based on results from the 4E setup (Fig. 2) is longer as compared to the cases of HP and NE (i.e., 10 ± 5 min, Table 2). In case of 4E measurements, the electrical current needs to transverse the skin membrane, which obviously is a different scenario as compared to the HP and NE measurements where the current travels mainly in the lateral direction. Thus, it is possible that the slower initial change of the 4E conductance is due to filling of voids in deeper regions of the SC, which naturally can be expected to require an even longer period. Still, the hydration times obtained with the HP, NE, and 4E methods are in surprisingly good agreement considering that these methodologies are quite different (Table 2).

So far, this discussion is primarily related to the conductance measurements performed with the different techniques. However, similar logics can be applied, not only to the conductance results, but also to the 1 kHz data and the data of the MIX parameter. In other words, the different impedance data show similar hydration responses, in qualitative terms, following the 16 min hydration protocol (Fig. 2). Importantly, the hydration times discussed here should not be considered as the time required to reach full hydration at thermodynamic equilibrium, which is likely to require a significantly longer time. Instead, as already proposed, the physical meaning of the hydration time is related to surface hydration and filling of voids of the superficial SC layers. Thus, the discussed hydration times are valid for the initial hydration process only, which is important to keep in mind. This general conclusion is in line with previous findings presented by Egawa and Tagami who used confocal Raman spectroscopy to investigate the changes of the water content of skin in vivo^46^. In brief, they concluded that even though there was a strong increase in the SC water content after 15 min of hydration, this change was mainly due to hydration of the most superficial regions of the SC^46^.

### In vivo−in vitro and cross-method correlations open up for simple in vitro testing

Detailed knowledge of the effect of hydration on the skin barrier properties is highly relevant for several reasons. For example, to take advantage of the occlusion effect for increased transdermal drug delivery it is crucial to characterize the time it takes to completely hydrate the skin barrier^10^. Similarly, this knowledge is important to consider for optimization of the extraction efficiency of LMW biomarkers from the skin, for example for early detection of skin cancer^2^, where elevated hydration is expected to increase the extraction^10^. Finally, for development of topical cosmetic formulations, it is relevant to study how the degree of skin hydration is affected by the formulation^19^. In fact, to support claims related to hydration and moisturization ascribed to topical cosmetic products, impedance-based measurements are commonly used^32^. Taken together, to facilitate this kind of research it is important to have safe, reliable, and simple in vitro tools that mimic the in vivo situation with high precision and accuracy. Pleasingly, based on the Spearman correlation plots in Fig. 4, it can be concluded that good correlations exist between in vivo and in vitro data obtained with the same methodology (Fig. 4 A–C). The fact that the results obtained from the in vitro experiments can be translated into the in vivo situation is promising as it opens up for simple, but accurate and precise, in vitro testing. In particular, in vitro impedance experiments can be performed under more controlled conditions, such as controlled external humidity and temperature, which otherwise is not always possible to control in case of in vivo measurements. Further, in vitro testing is more flexible in general and can easier accommodate specific conditions, which sometimes are required depending on the type of research question that is addressed.

In addition, as shown by the Spearman correlation plots in Fig. 5, it can be concluded that good correlations exist between the conductance data obtained with either the HP, NE, or the 4E methodologies (Fig. 5 A–D). This is an important finding as it implies that the effect of different parameters, such as application of cosmetic creams, on the skin hydration can be investigated with different methods and still arrive at the same conclusion. Even though good correlations are obtained in several occasions, it should be noted that the correlation plots are based on a limited number of replicates (*n* = 5–12), which is an important limitation to keep in mind.

### Prolonged hydration confirms that the hydration process occurs in two stages

The results from the prolonged skin membrane hydration study clearly show that the hydration process occurs in two distinct stages (Fig. 6). In the initial process, the impedance properties are changing abruptly. In contrast, the second hydration process results in more subtle changes of the impedance data, which proceeds at a slower rate and, seemingly, without any stable endpoint (Fig. 6). In line with these observations, previous in vivo studies employing confocal Raman microspectroscopy have shown that the water content profile across the skin barrier was significantly altered after 45 or 90 min of hydration, as compared to normal hydration^46,47^. However, even after 90 min of hydration, the water content profile was not observed to be completely homogeneous, indicating that no stable endpoint was reached^46,47^.

Speculatively, the impedance changes observed during the second hydration process are related to hydration induced structural changes of the SC lipid and protein molecular components, which are expected to proceed on a longer time scale^9–11,20,26^. It has been established that the molecular dynamics of lipids in the extracellular lamellae and amino acid residues of the keratin filaments are significantly increased by prolonged hydration, such as equilibration of SC samples in PBS solution or in a vapor phase with close to 100% relative humidity for 24 h^9,20,31^. Further, the corneocytes are known to swell significantly in thickness upon prolonged hydration (within hours), which implies that parts of the extracellular lipid domains surrounding the corneocytes have to undergo a drastic unfolding or realignment process to accommodate this dramatic swelling^27,28^. Taken together, the observed changes of the impedance properties during the second hydration process, occurring after about 1 h (Fig. 6), can be explained by hydration induced changes of the SC molecular components. These changes are expected to proceed continuously towards an equilibrium state where the chemical potential of water inside the SC tissue is equal to the corresponding value of the PBS medium. However, the equilibrium state is not reached within the limited experimental time used here (i.e., 3 h). In fact, it is likely that thermodynamic equilibrium will never be reached in this kind of experiments since the skin membrane is expected to disintegrate during prolonged hydration. For example, extended hydration exposure is associated with formation of macroscopically sized aqueous inclusions within the SC^23–25,29^. Since these aqueous pools do not appear instantaneously, the process of their formation may represent yet another explanation for the continuous changes of the impedance properties over extended hydration exposure.

### Temperature influences the dynamics of the initial and second hydration processes differently

The effect of temperature on the electrical impedance properties of the skin barrier has been studied previously^42–44^. In brief, it is well established that the skin membrane resistance decreases as a function of temperature and that the skin membrane capacitance increases in parallel^42–44^. At temperatures above approximately 60–70 °C, these electrical impedance parameters are observed to change dramatically^42–44^. This dramatic change is primarily due to thermotropic melting of the extracellular lipid lamellae, from a solid ordered state into a disordered liquid crystalline state^20,45^. However, the dynamic properties of the hydration process at different temperatures has, to the best of our knowledge, not been systematically investigated with respect to changes of the electrical impedance properties.

Strikingly, the dynamics of the initial hydration process was concluded to be more or less independent of the temperature; the estimated hydration times were 7 ± 1 min based on the 1 kHz data, 11 ± 2 min based on the conductance data, and 17 ± 2 min based on the MIX data (Table 3). This is an interesting conclusion which, speculatively, can be explained by that the initial hydration process is primarily related to filling of superficial voids of the skin barrier with electrolyte solution. If this is correct, it is reasonable to assume that this process is relatively independent of the temperature. Further, this conclusion implies that thermal effects, such as melting of lipids, occurs at longer time scales in a similar manner as compared to hydration induced changes of the biophysical properties of the SC molecular components^9,20,31^.

Turning to the temperature effect on the second hydration process, the results presented in Fig. 7 show that the rate of change, ∆ (s^−1^), of the impedance parameters between 60 and 180 min is affected by an elevated temperature in all cases. However, the response pattern of the skin conductance (Fig. 7A), skin resistance (Fig. 7B), and the MIX parameter (Fig. 7C) varies. Firstly, the rate of increase of the conductance (Fig. 7A) is observed to be constant between 12 and 42 °C and significantly increased at 52 °C and even more so at 72 °C. These observations imply that the conductive pathways remain more or less constant until the temperature reaches levels where thermally induced disorder of the extracellular SC lipid lamellae is expected^20,45^. This could introduce defective openings across the SC that are freely accessible for charge carriers.

A similar assertion can be used to explain the results of the skin resistance (Fig. 7B) and the MIX parameter (Fig. 7C). In brief, the rate of change of the skin resistance is observed to be constant at 12 and 22 °C, which implies that the SC barrier remains in a similar state at these temperatures (Fig. 7B). At higher temperatures, however, the rates of decrease of the resistance are growing linearly as a function of temperature (Fig. 7B). These observations imply that the charge transfer over longer distances (as compared to the conductance parameter that probes shorter distances) becomes less and less impeded. Interestingly, this linear dependence implies that the responsible mechanism should be ascribed to a process that changes the SC barrier properties in a continuous manner. Thus, it is not straightforward to assign this continuous change of the skin resistance to any thermotropic melting of the extracellular lipid lamellae^45^, which is expected to result in a more dramatic (non-linear) change. However, a previous study employing solid-state NMR on close to fully hydrated SC samples showed that the dynamical properties of the extracellular lipid lamellae continuously increased when the temperature increased stepwise from 32 to 40 °C, and finally to 60 °C^20^. In other words, it is not unlikely that thermally induced disorder of the SC lipids results in continuously more and more defective regions of the SC barrier where charge transfer can occur. In addition, the hydration process at elevated temperatures may increase the rate of corneocyte swelling and formation of water inclusions (disruption of the SC barrier)^23–25,29^, which is also expected to result in a decreased skin membrane resistance.

Finally, the MIX parameter is observed to be constant between 12 and 52 °C, while a distinct decrease is observed at 72 °C (Fig. 7C). This strongly suggest that thermal melting of SC extracellular lipid lamellar structures, occurring above 70 °C^20,45^, results in a significant decrease of the barrier properties of the SC (i.e., the 1 kHz frequency impedance decreases significantly more, in relative terms, as compared to the 10 kHz impedance)^42–44^.

## Conclusion

In this work we have employed three different methods to investigate the hydration process in a systematic manner by employing both in vivo and in vitro experiments. The major findings are:

- Based on in vivo and in vitro experiments with the hydration probe (HP) and the Nevisense (NE) methodologies, the hydration process is concluded to occur in two distinct stages with different rates of change of the electrical impedance response. This finding is supported by controlled in vitro experiments with the four electrode Franz cell (4E) method (Fig. 2).
- The initial hydration process, which is determined to occur within about 5–10 min depending on the method used to derive the hydration time, follows a first order decelerating kinetics behavior. This process is suggested to be due to filling of preexisting superficial air-filled voids of the SC (Fig. 3 and Table 2). The dynamics of this process is concluded to be independent of the temperature (Table 3). This conclusion supports that this process is mainly related to void filling and not to hydration induced alterations of the SC molecular components, which are expected to require longer equilibration times.
- Good correlation between both in vivo and in vitro measurements are observed (Fig. 4). Likewise, good correlation between conductance measurements obtained with HP, NE, and 4E are established (Fig. 5). These findings are promising as they open up for simple, but accurate and precise, in vitro testing with different impedance-based methods.
- The second hydration process, which is observed after about 60 min (Fig. 6), proceeds at a slower rate and in a linear manner. This process is suggested to be due to hydration induced alterations of the SC, such as alterations of the molecular structure and dynamics of SC lipids and proteins, swelling of the corneocytes, and/or formation of aqueous inclusions. The dynamics of the second hydration process are highly dependent on the temperature (Fig. 7), which lends further support to that the mechanism behind this process is related to alterations of the molecular structure and dynamics of SC molecular components (e.g., melting of SC lipids).

## Supplementary information

Supplementary Figures.
