# Supplementary material for: Skin hydration dynamics investigated by electrical impedance techniques in vivo and in vitro
Source: Sci Rep. 2020 Oct 14;10:17218. doi: 10.1038/s41598-020-73684-y (PMC7557913; doi:10.1038/s41598-020-73684-y)
Supplement: Supplementary file 1 — Supplementary Figures. [file 41598_2020_73684_MOESM1_ESM.docx]

# Skin hydration dynamics investigated by electrical impedance techniques *in vivo* and *in vitro*

Maxim Morin^1,2^, Tautgirdas Ruzgas^1,2^, Per Svedenhag^3^, Christopher D., Anderson^4^, Stig Ollmar^5^, Johan Engblom^1,2^, and Sebastian Björklund^1,2,^*

^1^Biofilms – Research Center for Biointerfaces, Malmö University, Sweden

^2^Department of Biomedical Science, Faculty of Health and Society, Malmö University, Malmö, Sweden

^3^SciBase AB, Sundbyberg, Sweden

^4^Department of Biomedical and Clinical Sciences, Linköping University, Linköping, Sweden

^5^Department of Clinical Science, Intervention and Technology, Karolinska Institutet, Stockholm, Sweden

*Corresponding author: sebastian.bjorklund@mau.se

Figure S1. Representative results from two-sided t-tests comparing the mean values (±SD) of the relative change of the electrical impedance (EI) data obtained with the NE instruments *in vivo* from different individuals (P1, P2. P3, P4; *n* = 3 for each individual) and different hydration regions (R1, R2, R3; *n* = 4 for each hydration region). The relative change refers to the change of the EI data between 5 seconds and 15 minutes. As shown, the relative change of the EI data from different individuals (P) are similar as compared to the corresponding data obtained from skin regions (R) on the same individual. By performing two-sided t-tests for comparing the mean values of the different data sets, with the total average value (*n* = 12), no evidence for any statistically significant differences could be established. In fact, from a systematic evaluation, this conclusion is also valid for two-sided t-tests comparing the mean values, both within groups and between groups, irrespective of impedance method used to collect the data or if the experiments were performed *in vivo* or *in vitro*. Based on this conclusion, it is reasonable to treat each hydration series as one individual replicate, irrespective of the origin of the hydration region, which can be either from the same subject (or ear) or from different subjects (or ears).

Figure S2. Nyquist plot of impedance data.

| (A) Bode plot of NE impedance data | (B) Bode plot of 4E impedance data |
| --- | --- |
| 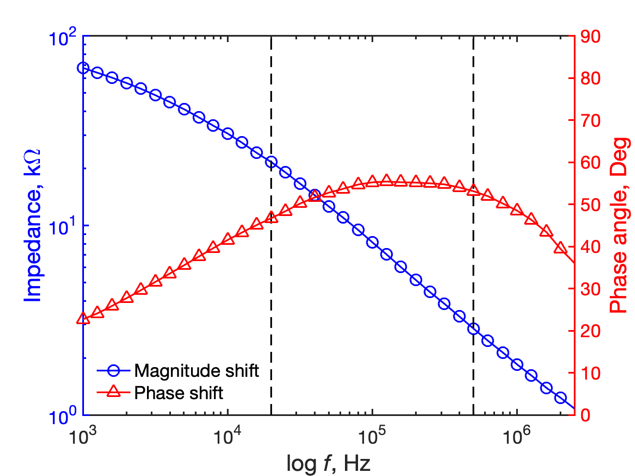 | 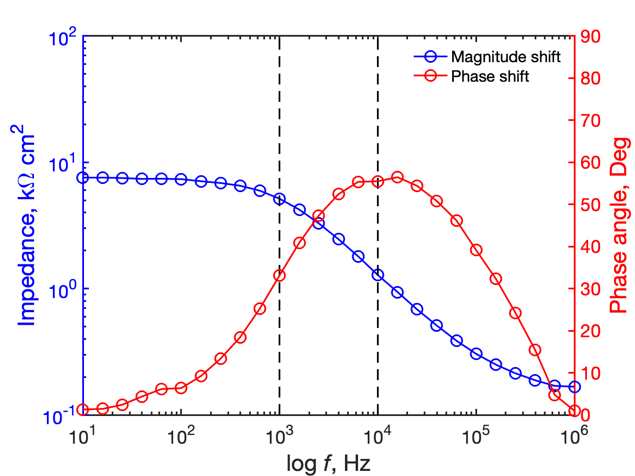 |
| Figure S3. Bode plots showing (A) skin impedance measured with NE between 1 kHz and 2.5 MHz and (B) skin impedance measured with 4E between 10 Hz and 1 MHz. The magnitude and phase shifts are shown as blue and red curves, respectively. Black dashed lines show frequencies used for determination of MIX indices (NE: 20 kHz vs 500 kHz; 4E: 1 kHz vs 10 kHz). | |
